# Comorbid obstructive sleep apnea is associated with adverse cardiovascular outcomes in female patients with acute coronary syndrome complicating metabolic syndrome

**DOI:** 10.1002/clc.24020

**Published:** 2023-04-14

**Authors:** Bin Wang, Xiaogang Liu, Wen Hao, Jingyao Fan, Bin Que, Hui Ai, Xiao Wang, Shaoping Nie

**Affiliations:** ^1^ Division of Cardiology, Center for Coronary Artery Disease, Beijing Anzhen Hospital Capital Medical University Beijing China; ^2^ National Clinical Research Center for Cardiovascular Diseases Beijing China; ^3^ Department of Cardiology Wuhan Fourth Hospital Wuhan China

**Keywords:** acute coronary syndrome, metabolic syndrome, obstructive sleep apnea

## Abstract

**Background:**

Obstructive sleep apnea (OSA) and metabolic syndrome (MetS) are each increasingly common in patients with acute coronary syndrome (ACS). Whether OSA increases cardiovascular consequences in ACS patients with MetS has not been investigated.

**Hypothesis:**

OSA increases cardiovascular risk in ACS patients with MetS. We aimed to examine the association between OSA and cardiovascular consequences in ACS patients with MetS.

**Methods:**

In this prospective cohort study, we consecutive recruited 2160 ACS patients who underwent portable sleep breathing monitoring. OSA is defined as an apnea‐hypopnea index (AHI) ≥ 15 events/h. The primary endpoint was major adverse cardiovascular and cerebrovascular events (MACCE), including cardiovascular death, myocardial infarction, stroke, ischemia‐driven revascularization, or hospitalization for unstable angina or heart failure.

**Results:**

A total of 1927 patients with ACS were enrolled. Among them, 1486 (77.1%) had MetS and 1014 (52.6%) had OSA. During 2.9 years of follow‐up, the cumulative incidence of MACCE was similar between OSA and non‐OSA groups in patients with MetS (21.9% vs. 17.9%, adjusted hazard ratio [HR] = 1.29 95% confidence interval [CI]: 0.99–1.67, *p* = .06) and patients without MetS (24.4% vs. 17.3%, adjusted HR = 1.21 95% CI: 0.73–2.03, *p* = .46). Patients with MetS and OSA had a significantly higher risk of MACCE than patients with MetS and without OSA in women (27.8% vs. 18.1%, adjusted HR = 1.70, 95% CI: 1.01–3.09, *p* = .04) but not in men (21.0% vs. 17.9%, adjusted HR = 1.19, 95% CI: 0.91–1.59, *p* = .21).

**Conclusions:**

In hospitalized ACS patients with MetS, comorbid OSA was associated with increased risk of cardiovascular consequences among women.

## Graphical abstract

This prospective cohort study included 1927 hospitalized acute coronary syndrome (ACS) patients from 2015 to 2020. Among them, 1486 (77.1%) had MetS and 1014 (52.6%) had obstructive sleep apnea (OSA). At a median follow‐up of 2.9 years, OSA was associated with a greater risk of cardiovascular events in female ACS patients with MetS (27.8% vs. 18.1%, adjusted hazard ratio [HR] = 1.70, 95% confidence interval [CI]: 1.01–3.09, *p* = .04) but not in men (21.0% vs. 17.9%, adjusted HR = 1.19, 95% CI: 0.91–1.59, *p* = .21). In conclusion, female ACS patients with MetS should not be neglected for OSA screening and dedicated intervention studies focusing on women ACS patients with MetS and comorbid OSA should be prioritized.

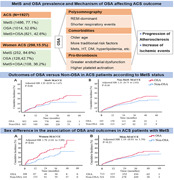

## 1 INTRODUCTION

Obstructive sleep apnea (OSA) and metabolic syndrome (MetS) are each increasingly common in patients with acute coronary syndrome (ACS), and the relationship between OSA and MetS is unequivocal and bidirectional.^1^, ^2^ OSA is associated with oxidative stress, systemic inflammation, sympathetic activation, endothelial dysfunction, and visceral fat dysfunction that could induce or exacerbate metabolic disorders, including insulin resistance, hypertension, central obesity, and dyslipidemia.^3^, ^4^, ^5^, ^6^ All these are components of MetS, which form a group of metabolic abnormalities, including hypertriglyceridemia, hypertension, impaired glucose metabolism, central obesity, and low high‐density lipoprotein cholesterol (HDL‐C).^7^ Moreover, current studies have identified several metabolic disturbances that cause and exacerbate OSA, the mechanisms include anatomical and neurological effects on the upper airway.^8^, ^9^

Existing data have suggested that the pathophysiology mechanisms of OSA and MetS interact with each other and that both have adverse effects on the prognosis of patients with ACS.^10^, ^11^, ^12^, ^13^, ^14^, ^15^ In addition, both OSA and MetS have gender differences in the pathogenesis, clinical symptoms, prevalence of comorbidities, and cardiovascular consequences.^16^, ^17^ However, whether OSA increases the risk of recurrent cardiovascular events in ACS patients with MetS has not been directly investigated. Therefore, based on a large prospective cohort study, we elucidated the association between OSA and subsequent cardiovascular events in ACS patients according to MetS and stratified by gender.

## 2 METHODS

### 2.1 Study design and participants

This is an auxiliary study of OSA–ACS study (NCT03362385), a large, prospective study designed to elucidate the association between OSA and subsequent cardiovascular events of hospitalized ACS patients. This study protocol has been described in previous studies.^12^, ^18^ Briefly, we consecutively recruited ACS patients aged 18–85 years and underwent portable sleep breathing monitoring from June 2015 to January 2020 at Beijing Anzhen Hospital, Capital Medical University. Exclusion criteria: cardiac arrest or cardiogenic shock, malignancy, and sleep study failure. Then, patients with predominantly central sleep apnea (≥50% central events and a central AHI ≥ 10/h), recording time < 180 min, and patients treated with continuous positive airway pressure (CPAP) therapy (>4 h/day and >21days/month) after discharge were excluded. In the final analysis, patients who lost follow‐up were also excluded from the analysis.

This study conformed to the STROBE (Strengthening the Reporting of Observational studies in Epidemiology) guidelines and was conducted in accordance with the Declaration of Helsinki.^19^, ^20^ The Ethics Committee of Beijing Anzhen Hospital, Capital Medical University approved this study (2013025). All patients provided written informed consent.

### 2.2 MetS diagnosis

The criteria for diagnosis of MetS are three or more risk factors based on the criteria established in the Joint Scientific Statement^21^: (1) Elevated waist circumference ≥90 cm for males and ≥80 cm for females; (2) triglycerides ≥ 1.7 mmol/L, or treatment for elevated triglycerides; (3) HDL‐C < 1.0 mmol/L in males, <1.3 mmol/L in females, or treatment for HDL‐C; (4) systolic blood pressure (SBP) ≥ 130 mmHg and/or diastolic blood pressure (DBP) ≥ 85 mmHg, or with a history of treatment for hypertension; and (5) fasting blood glucose level ≥ 5.6 mmol/L or treatment of elevated glucose.

### 2.3 Overnight sleep study

All patients underwent an overnight sleep study after clinical stabilization during hospitalization by a type III portable sleep monitoring device (ApneaLink Air). The following signals were recorded: nasal airflow, thoraco‐abdominal movements, snoring episodes, pulse, and oxygen saturation (SaO_2_). Sleep data were manually scored by two sleep investigators, who were blinded to patients' demographic and clinical characteristics. When the results were inconsistent, they were determined by a senior sleep physician. Sleep data were scored and defined according to the standards of the American Academy of Sleep Medicine (2007). Apneas were defined as the complete cessation of airflow lasting 10 s or more. Hypopnea was defined as a 30% reduction in airflow for lasting ≥ 10 s accompanied by an oxygen desaturation of ≥ 4%. The AHI was computed as the number of episodes of apnea and hypopnea per hour of recording. The oxygen saturation index (ODI) was calculated as the number of times per hour of sleep that oxygen saturation decreased by 4% or more from baseline. Excessive daytime sleepiness (EDS) was measured by Epworth Sleepiness Scale (ESS). Study participants with AHI ≥ 15 events/h were considered as the OSA group, AHI < 15 events/h were considered as the non‐OSA group.^18^

### 2.4 Procedures and management

All patients received standard medical care in line with current clinical practice guidelines.^22^, ^23^ At discharge, all patients were receiving aspirin (100 mg daily) plus clopidogrel (75 mg daily) or ticagrelor (90 mg twice daily) for at least 1 year unless clinically contraindicated. Participants who had a diagnosis of moderate‐to‐severe OSA (AHI ≥ 15), particularly those with severe excessive daytime sleepiness, were referred to the sleep centers for further evaluation.

### 2.5 Follow‐up and outcomes

Patients' follow‐up visits were scheduled at baseline,1, 3, 6, and 12 months and then every 6 months thereafter. Clinical cardiovascular and cerebrovascular events were evaluated with data collected during clinic visits, telephone calls, or medical record review by investigators who was blinded to the patients' results of the sleep study. Recurrent cardiovascular events were recorded at each follow‐up visit and adjudicated by an independent clinical event committee.

All endpoints were defined per standardized cardiovascular trial protocol.^24^ A composite of the major adverse cardiovascular and cerebrovascular events (MACCE) is the primary outcome for our study, defined as a composite of cardiovascular death, myocardial infarction (MI), stroke, ischemic‐driven revascularization, or hospitalization for unstable angina or heart failure. Other outcomes included the 6 individual components of the primary outcome, also all‐cause death, all repeat revascularization, a composite of major adverse cardiovascular events (including cardiovascular death, nonfatal myocardial infarction, and stroke), and a composite of adverse cardiac events (cardiovascular death, ischemia‐driven revascularization, nonfatal MI, unstable angina, or hospitalization for heart failure).^18^

### 2.6 Statistical analyses

We used counts and percentages (%) to describe qualitative variables and mean ± SD or medians with interquartile ranges (IQRs) for continuous variables. Categorical variables were analyzed by *χ* ^2^ or Fisher's exact tests, as appropriate. Continuous variables, where appropriate, were compared by an unpaired *t*‐test or Mann–Whitney *U* test. Kaplan–Meier survival analysis was performed by log‐rank test. Hazard ratios (HR) and 95% confidence interval (95% CI) were calculated with the fully adjusted Cox proportional hazard regression models, which included age, gender, body mass index, smoking, hypertension, diabetes mellitus, hyperlipidemia, previous myocardial infarction, left ventricular ejection fraction, and clinical manifestations (unstable angina vs. acute myocardial infarction). For patients who experienced more than one adverse outcome, only the first adverse event was considered for this analysis. A multiplicative interaction term was added to the adjusted model to determine whether the association between OSA and the risk of cardiovascular events was modified by sex in patients with MetS. All statistical analyses were calculated by SPSS V.26.0 (IBM SPSS). A two‐sided *p* < .05 was considered statistically significant.

## 3 RESULTS

### 3.1 Baseline characteristics

**Figure 1:**
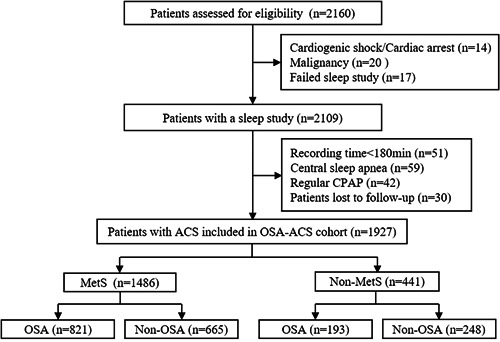
Study flowchart. ACS, acute coronary syndrome; CPAP, continuous positive airway pressure; MetS, metabolism syndrome; OSA, obstructive sleep apnea.

A total of 1927 patients with ACS were enrolled (Figure 1) aged (56.4 ± 10.5) years. Among them, 1486 (77.1%) patients had MetS, and 1014 (52.6%) had OSA. Patients with OSA were more likely to be male, and exhibited higher glycated hemoglobin levels, body mass index, waist circumference, and waist‐to‐hip ratio. In the MetS group, patients with OSA have a higher proportion of traditional risk factors, including hypertension, diabetes mellitus, diagnosis of myocardial infarction, PCI procedures, and drug‐eluting stents, and are more likely to receive antihypertensive drugs. Among patients without MetS, those with OSA are more likely to have had previous PCI and have a poorer left ventricular function. Other information was generally well matched between patients with and without OSA, regardless of MetS status. The detailed baseline characteristics by MetS and OSA categories are shown in Table 1. Baseline data stratified according to gender and OSA/MetS status are presented in Tables S1 and S2.

**Table 1:** Baseline clinical characteristics in ACS patients according to MetS status.

Variables	Total (*n* = 1927)	MetS (*n* = 1486)			Non‐MetS (*n* = 441)		
		OSA (*n* = 821)	Non‐OSA (*n* = 665)	*p* value	OSA (*n* = 193)	Non‐OSA (*n* = 248)	*p* value
Demographics							
Age, y	56.4 ± 10.5	56.1 ± 10.5	56.0 ± 10.3	.80	58.3 ± 10.5	56.9 ± 10.6	.17
Male	1629 (84.5)	713 (86.8)	521 (78.3)	<.001	173 (89.6)	222 (89.5)	.97
BMI, kg/m^2^	27.1 ± 3.6	27.7 ± 3.8	26.2 ± 3.81	.002	26.6 ± 3.9	24.4 ± 3.1	<.001
Waist	99.5 ± 9.6	103.1 ± 8.8	98.6 ± 8.1	<.001	97.6 ± 10.8	91.1 ± 8.6	<.001
Neck, circumference, cm	41 (38–43)	37 (36–39)	36 (34–39)	.44	40 (38–42)	38 (37–40)	<.001
Waist‐to‐hip ratio, median	0.98 (0.95–1.02)	0.99 (0.96–1.03)	0.98 (0.94–1.01)	<.001	0.97 (0.94–1.00)	0.94 (0.91–0.99)	<.001
Systolic BP, mm Hg	126 (117–138)	130 (120–142)	130 (120–144)	.74	120 (110–130)	121 (112–133)	.44
Diastolic BP, mm Hg	76 (70–84)	71 (69–80)	74 (68–80)	.50	72 (68–80)	72 (67–80)	.99
Medical history							
Diabetes mellitus	609 (31.6)	295 (35.9)	270 (40.6)	.07	24 (12.4)	20 (8.1)	.13
Hypertension	1247 (64.7)	613 (74.7)	454 (68.3)	.006	78 (40.4)	102 (41.1)	.88
Hyperlipidemia	637 (33.1)	298 (36.3)	232 (34.9)	.57	45 (23.3)	62 (25.0)	.68
Prior stroke	207 (10.7)	103 (12.5)	63 (9.5)	.06	18 (9.3)	23 (9.3)	.99
Prior MI	316 (16.4)	148 (18.0)	105 (15.8)	.25	29 (15.0)	34 (13.7)	.70
Previous PCI	399 (20.7)	197 (24.0)	135 (20.3)	.09	37 (19.2)	30 (12.1)	.04
Previous CABG	29 (1.5)	16 (1.9)	11 (1.7)	.67	2 (1.0)	0 (0)	.11
Smoking				.52			.34
No	654 (33.9)	284 (34.6)	243 (36.5)		49 (25.4)	78 (31.5)	
Current	913 (47.4)	390 (47.5)	296 (44.5)		106 (54.9)	121 (48.8)	
Previous	360 (18.7)	147 (17.9%)	126 (18.9)		38 (19.7)	49 (19.8)	
Baseline tests							
Glucose, mmol/L	6.0 (5.3–7.5)	6.5 (5.7–8.4)	6.1 (5.5–7.6)	.05	5.3 (5.0–5.8)	5.3 (5.0–5.7)	.36
Hemoglobin A1C, %	6.1 (5.6–7.0)	6.8 (6.1–8.2)	6.4 (5.9–7.4)	.01	5.8 (5.4–6.1)	5.4 (5.4–6.0)	.04
Triglyceride, mmol/L	1.5 (1.1–2.2)	1.6 (1.2–2.1)	1.6 (1.2–2.2)	.22	1.2 (0.9–1.4)	1.1 (0.8–1.4)	.09
Total Cholesterol, mmol/L	4.1 (3.5–4.9)	4.3 (3.6–5.2)	4.5 (3.6–5.2)	.81	4.2 (3.6–4.8)	4.0 (3.4–4.8)	.15
HDL‐C, mmol/L	1.0 (0.9–1.2)	1.1 (0.9–1.2)	1.1 (1.0–1.3)	.59	1.1 (1.0–1.3)	1.2 (1.0–1.3)	.91
LDL‐C, mmol/L	2.4 (1.9–3.1)	2.4 (2.0–3.1)	2.6 (1.9–3.2)	.89	2.6 (2.1–3.1)	2.4 (1.8–3.2)	.12
Cr, μmmol/L	73.7 (64.8–83.9)	63.4 (55.7–74.6)	59.7 (52.1–70.3)	.13	74.3 (63.9–84.3)	72.7 (64.0–81.7)	.19
LVEF, %	61 (56–65)	63 (59–68)	63 (60–66)	.86	60 (54–65)	62 (56–66)	.03
Diagnosis				.02			.50
STEMI	430 (22.3)	197 (24.0)	120 (18.0)		54 (28.0)	59 (23.8)	
NSTEMI	365 (18.9)	155 (18.9)	131 (18.7)		36 (18.7)	43 (17.3)	
Unstable angina	1132 (58.7)	469 (57.1)	414 (62.3)		103 (53.4)	146 (58.9)	
Sleep study							
AHI, events/h	16.0 (8.0–30.0)	29.5 (21.2–42.8)	7.8 (4.4–11.0)	<.001	27.4 (19.8–39.2)	7.2 (3.4–10.2)	<.001
ODI, events/h	16.2 (8.8–28.6)	27.9 (20.5–40.3)	8.8 (5.0–12.0)	<.001	24.9 (18.1–36.4)	8.1 (4.4–11.3)	<.001
Minimum SaO_2_, %	85 (81–88)	82 (77–86)	87 (84–89)	<.001	84 (78–87)	88 (86–90)	<.001
Mean SaO_2_, %	94 (93–95)	93 (92–94)	94 (93–95)	<.001	93 (92–95)	95 (94–95)	<.001
Time with SaO_2_ < 90%	2.3 (0.4–10.0)	7.0 (2.0–17.0)	1 (0.1–3.0)	<.001	4 (1–13)	0.4 (0.0–1.4)	<.001
Epworth Sleepiness Scale	7.0 (4.0–11.0)	8.6 (5.0–12.0)	6.9 (3.0–10.0)	<.001	7.4 (3.0–11.0)	7.0 (3.0–10.0)	.61
Procedures							
Coronary angiography	1877 (97.4)	802 (97.7)	645 (97.0)	.41	188 (97.4)	242 (97.6)	.91
PCI	1209 (62.7)	541 (65.9)	393 (59.1)	.007	126 (65.3)	149 (60.1)	.26
DES use	1051 (86.9)	469 (57.1)	341 (51.3)	.02	112 (58.0)	129 (52.0)	.21
CABG	130 (6.7)	50 (6.1)	55 (8.3)	.10	9 (4.7)	16 (6.5)	.42
Medications on discharge							
Aspirin	1877 (97.4)	797 (97.1)	646 (97.1)	.94	190 (98.4)	244 (98.4)	.96
P2Y_12_ inhibitors	1768 (91.7)	759 (92.4)	599 (90.1)	.11	179 (92.7)	231 (93.4)	.87
β‐blockers	1488 (77.2)	665 (79.8)	516 (77.6)	.31	144 (74.6)	173 (69.8)	.26
ACEIs/ARBs	1195 (62.0)	558 (68.0)	400 (60.2%)	.002	107 (55.4)	130 (52.4)	.53
Statins	1897 (98.4)	805 (98.1)	656 (98.6)	.38	192 (99.5)	244 (98.4)	.28

*Note*: Data are presented as mean ± SD, median (IQR), *n* (%), or *n* (%).

Abbreviations: ACEI, angiotensin‐converting enzymes inhibitor; AHI, apnea‐hypopnea index; ARB, angiotensin receptor blocker; BMI, body mass index; BP, blood pressure; CABG, coronary artery bypass grafting; Cr, creatinine; DES, drug‐eluting stent; IQR, interquartile range; LVEF, left ventricular ejection fraction; MetS, metabolism syndrome; NSTEMI, non‐ST‐segment elevation myocardial infarction; ODI, oxygen desaturation index; OSA, obstructive sleep apnea; PCI, percutaneous coronary intervention; SD, standard deviation; STEMI, ST‐segment‐elevation myocardial infarction.

### 3.2 Results of sleep study

The mean overall total sleep time was 472 (405–536) min. The AHI recorded in the study ranged from 0 to 97.9. According to AHI ≥ 15, the prevalence of OSA was 55.2% in all patients. The prevalence of OSA in MetS patients was higher than that in non‐MetS patients (55.2% vs. 43.8%; *p* < .001). Minimum oxygen saturation in patients with OSA was lower than those in patients with non‐OSA. The detailed sequence information of sleep is listed in Table 1.

### 3.3 Outcomes by MetS

During a median follow‐up 2.9 (1.5–3.6) years, the primary outcome of MACCE occurred in 389 of 1927 patients (20.2%). There were no statistically significant differences in the cumulative incidence of MACCE between MetS and non‐MetS group (20.1% vs. 20.4%, adjusted HR = 0.88, 95% CI: 0.68–1.14, *p* = .33); the individual endpoints of cardiovascular death, MI, stroke, ischemia‐driven revascularization, hospitalization for unstable angina or heart failure; the composite of cardiovascular death, MI, or ischemic stroke; and the composite of a cardiac event (Figure S1 and Table S3). The crude numbers of events between MetS and non‐MetS are listed in Table S4.

### 3.4 Outcomes of OSA versus non‐OSA groups by MetS and by sex

The cumulative incidence of MACCE was similar between OSA and non‐OSA groups in patients with MetS (21.9% vs. 17.9%, adjusted HR = 1.29 95% CI: 0.99–1.67, *p* = .06) and patients without MetS (24.4% vs. 17.3%, adjusted HR = 1.21 95% CI: 0.73–2.03, *p* = .46). (Figure S2 and Table 2). There was no significant difference in the incidence of the individual endpoints of myocardial infarction, cardiovascular death, ischemic stroke, ischemia‐induced revascularization, hospitalization for unstable angina, and hospitalization for heart failure; the composite of myocardial infarction, cardiovascular death, or ischemic stroke; the composite endpoint cardiac events between OSA and non‐OSA group in both patients with MetS and non‐MetS (Table 2).

**Table 2:** Clinical outcomes in OSA versus non‐OSA groups according to MetS status.

Variables	Unadjusted HR (95% CI)	*p* value	Adjusted HR^a^ (95% CI)	*p* value
MACCE				
MetS	1.32 (1.04–1.66)	.02	1.29 (0.99–1.67)	.06
Non‐MetS	1.44 (0.95–2.17)	.09	1.21 (0.73–2.03)	.46
Cardiovascular death				
MetS	1.42 (0.65–3.11)	.38	1.04 (0.45–2.44)	.92
Non‐MetS	0.63 (0.12–3.43)	.59	0.10 (0.01–2.25)	.15
Myocardial infarction				
MetS	1.63 (0.85–3.11)	.14	1.42 (0.68–2.999)	.35
Non‐MetS	1.85 (0.52–6.56)	.34	0.50 (0.08–3.24)	.47
Stroke				
MetS	1.15 (0.58–2.30)	.69	1.27 (0.58–2.78)	.56
Non‐MetS	1.94 (0.55–6.87)	.31	3.68 (0.64–21.30)	.15
Ischemia‐driven revascularization				
MetS	1.37 (0.95–1.97)	.09	1.44 (0.94–2.19)	.09
Non‐MetS	1.30 (0.68–2.50)	.43	0.80 (0.36–1.78)	.58
Hospitalization for unstable angina				
MetS	1.23 (0.93–1.62)	.14	1.25 (0.92–1.70)	.16
Non‐MetS	1.63 (0.83–2.23)	.22	1.13 (0.62–2.04)	.69
Hospitalization for heart failure				
MetS	0.65 (0.24–1.76)	.40	0.40 (0.13–1.20)	.40
Non‐MetS^b^	4.83 (0.54–43.3)	.16	‐	‐
Composite for cardiovascular death, myocardial infarction, or ischemic stroke				
MetS	1.11 (0.92–1.46)	.06	1.40 (0.88–2.23)	.16
Non‐MetS	1.60 (0.72–3.52)	.25	1.19 (0.40–3.58)	.75
Composite for cardiac events				
MetS	1.50 (0.99–2.28)	.06	1.26 (0.96–1.65)	.10
Non‐MetS	1.37 (0.88–2.12)	.16	1.09 (0.63–1.87)	.76
All‐cause death				
MetS	1.05 (0.55–2.03)	.88	0.92 (0.44–1.94)	.83
Non‐MetS	0.54 (0.14–2.10)	.38	0.54 (0.25–2.76)	.06
All repeat revascularization				
MetS	1.15 (0.85–1.54)	.36	1.15 (0.81–1.61)	.44
Non‐MetS	1.47 (0.84–2.58)	.18	1.26 (0.67–2.42)	.49

*Note*: Data are presented as median (IQR).

Abbreviations: CI, confidence interval; HR, hazard ratio; MACCE, major adverse cardiovascular and cerebrovascular event; MetS, metabolism syndrome; OSA, obstructive sleep apnea.

a Model adjusted for age, sex, body mass index, smoking, hypertension, diabetes mellitus, hyperlipidemia, prior myocardial infarction, prior stroke, left ventricular ejection fraction, and clinical presentation (acute myocardial infarction vs. unstable angina).

^b^ Univariate and/or multivariate Cox regression was not done due to no or few number of events. Composite for cardiac events (cardiovascular death, myocardial infarction, ischemia‐driven revascularization, or hospitalization for unstable angina or heart failure).

**Figure 2:**
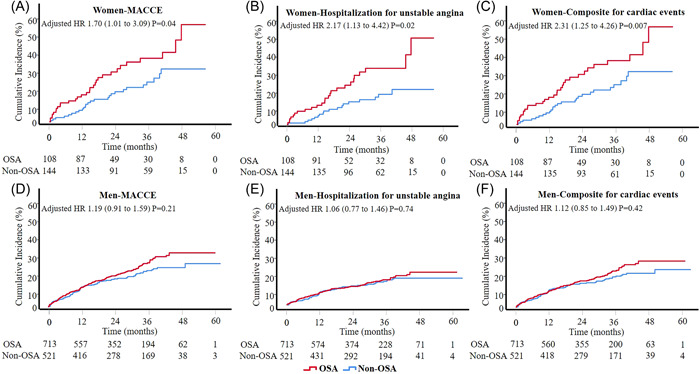
Cumulative incidence of hospitalization for MACCE, unstable angina, and composite for cardiac events by sex and OSA categories in ACS patients with MetS. Kaplan–Meier estimates and fully‐adjusted HR for hospitalization for MACCE (A, D), unstable angina (B, E), and Composite for cardiac events (C, F) between OSA and non‐OSA groups in women (A, B, C) and men (D, E, F). Composite for cardiac events (cardiovascular death, myocardial infarction ischemia‐driven revascularization, or hospitalization for unstable angina or heart failure); HR, hazard ratio; OSA, obstructive sleep apnea.

In a sex‐stratified analysis, for patients with MetS, the presence of OSA was associated with a higher incidence of MACCE compared with patients without OSA in women (27.8% vs. 18.1%, adjusted HR = 1.70, 95% CI: 1.01–3.09, *p* = .04) (Figure 2A), ‐but not in men (21.0% vs. 17.9%, adjusted HR = 1.19, 95% CI: 0.91–1.59, *p* = .21) (Figure 2D). The incremental risk in women was mainly driven by hospitalization for unstable angina (23.1% vs. 12.5%, adjusted HR = 2.17, 95% CI: 1.13–4.42, *p* = .02) (Figure 2B). Additionally, patients with OSA also had a higher incidence of composite rate of cardiac events compared with patients with non‐OSA in women (25.9% vs. 15.3%, adjusted HR = 2.31 95% CI: 1.25–4.26, *p* = .007) (Figure 2C) but not in men (19.1% vs. 16.7%, adjusted HR = 1.12 95% CI: 0.85–1.49, *p* = .42) (Figure 2F and Table 3). There was no interaction between gender in MACCE and OSA (interaction *p* = .29). The crude numbers of events by Sex and OSA Categories in ACS patients with MetS are listed in Table S5. Among patients without MetS, compared with non‐OSA, the presence of OSA has a similar risk of MAACE in women (30.0% vs. 23.1%, adjusted HR = 1.78 95% CI: 0.43–7.32, *p* = .43) and men (23.7% vs. 16.7%, adjusted HR = 1.37 95% CI: 0.83–2.25, *p* = .22) (Table S6). The crude number of all events in ACS patients without MetS by Sex and OSA Categories are listed in Table S7.

**Table 3:** Cox regression analyses evaluating the association between OSA and risk of cardiovascular events by sex in patients with ACS and MetS.

	Women (*n* = 252) OSA (*n* = 108) non‐OSA (*n* = 144)				Men (*n* = 1234) OSA (*n* = 713) non‐OSA (*n* = 521)			
Variables	Unadjusted HR (95% CI)	*p* value	Adjusted HR^a^ (95% CI)	*p* value	Unadjusted HR (95% CI)	*p* value	Adjusted HR^a^ (95% CI)	*p* value
MACCE	1.80 (1.06–3.04)	.03	1.70 (1.01–3.09)	.04	1.24 (0.96–1.61)	.10	1.19 (0.91–1.59)	.21
Cardiovascular death	2.81 (0.25–31.04)	.40	3.35 (0.27–42.04)	.35	1.25 (0.55–2.86)	.59	1.19 (0.51–2.79)	.70
Myocardial infarction	2.82 (0.52–15.40)	.23	2.93 (0.47–18.34)	.25	1.43 (0.71–2.88)	.31	1.42 (0.67–2.98)	.36
Stroke	0.44 (0.09–2.23)	.32	0.28 (0.05–1.51)	.14	1.64 (0.71–3.80)	.25	1.66 (0.70–3.96)	.25
Ischemia‐driven revascularization	1.91 (0.81–4.54)	.14	2.35 (0.91–6.04)	.08	1.27 (0.85–1.90)	.24	1.20 (0.79–1.83)	.39
Hospitalization for unstable angina	2.19 (1.19–4.01)	.01	2.17 (1.13–4.42)	.02	1.10 (0.81–1.50)	.56	1.06 (0.77–1.46)	.74
Hospitalization for heart failure^b^	‐	‐	‐	‐	0.74 (0.26–2.12)	.58	0.64 (0.22–1.89)	.42
Composite for cardiovascular death, myocardial infarction, or ischemic stroke	1.23 (0.47–3.18)	.67	1.58 (0.55–4.54)	.39	1.58 (0.99–2.53)	.06	1.51 (0.92–2.45)	.10
Composite for cardiac events	2.00 (1.17–3.57)	.01	2.31 (1.25–4.26)	.007	1.20 (0.91–1.57)	.19	1.12 (0.85–1.49)	.42
All‐cause death	1.41 (0.35–5.63)	.63	0.40 (0.10–1.61)	.20	1.01 (0.48–2.13)	.99	0.86 (0.43–2.02)	.93
All repeat revascularization	2.17 (0.97–4.83)	.06	2.74 (1.15–6.71)	.02	1.01 (0.73–1.39)	.97	0.95 (0.68–1.33)	.78

*Note*: Data are presented as median (IQR).

Abbreviations: CI, confidence interval; HR, hazard ratio; MACCE, major adverse cardiovascular and cerebrovascular event; MetS, metabolism syndrome; OSA, obstructive sleep apnea.

a Model adjusted for age, sex, body mass index, smoking, hypertension, diabetes mellitus, hyperlipidemia, prior myocardial infarction, prior stroke, and clinical presentation (acute myocardial infarction vs. unstable angina).

^b^ Univariate and multivariate Cox regression was not done due to few numbers of events; composite for cardiac events (cardiovascular death, myocardial infarction, ischemia‐driven revascularization, or hospitalization for unstable angina or heart failure).

## 4 DISCUSSIONS

To our knowledge, this is the first study to address the effect of comorbid OSA and MetS on cardiovascular outcomes in patients with established ACS. We found that ACS patients with MetS with comorbid OSA were associated with an increased risk of cardiovascular events in women. The increased risk associated with OSA in women might be explained by cardiac adverse events.

MetS, as a cluster of abdominal obesity, hypertriglyceridemia, hypertension, or low HDL cholesterol, and impaired glucose metabolism, is strongly associated with increased cardiovascular morbidity and mortality.^7^, ^11^ A meta‐analysis evaluating the interaction between MetS and cardiovascular clinical outcomes showed that the presence of MetS resulted in a 78% increase in overall cardiovascular risk.^25^ However, we found that the long‐term clinical outcomes of patients with ACS were not affected by MetS. Several previous studies have similar results that MetS has no impact on the clinical outcomes of ACS patients.^26^ The advanced technology of drug‐eluting stent and standardized drug therapy may partly explain this phenomenon. The progression of MetS to overt cardiovascular disease is multifactorial and complex and may be linked to visceral fat, insulin resistance, inflammatory responses, and sympathetic activation.^7^ Emerging evidence has demonstrated that MetS is considered a major risk factor for the development and progression of OSA.^2^ Additionally, OSA has itself been linked to an increased likelihood of adverse cardiovascular outcomes following ACS.^12^, ^18^ In terms of mechanisms, OSA primarily stimulates a variety of different mechanisms through intermittent hypoxia, including sympathetic activation, inflammation, oxidative stress, and endothelial dysfunction that are detrimental to the cardiovascular system.^5^, ^27^ Furthermore, the Sleep and Stent Study, which designed evaluating the effects of OSA on cardiovascular outcomes in patients undergoing PCI, found that the combination of OSA and DM is a strong risk marker for the occurrence of MACCE after PCI.^28^ Similarly, it is will be crucial to investigate whether OSA would have any additional excitatory effect on cardiovascular events in ACS patients with MetS. In the present study, we found that ACS patients with MetS and OSA were not associated with an increased risk of MACCE than patients with MetS without OSA.

These negative results might be explained by gender differences. Previous studies have shown that ACS has gender differences in coronary artery anatomy, baseline risk factors, symptoms, treatment effects, comorbidities, and outcomes.^29^ A recent intravascular imaging study showed differences between women and men, with women exhibiting coronary plaque features such as reduced overall plaque burden, calcification, and reduced signs of necrosis in the plaque core.^30^ In addition, for ACS patients, gender also has different effects on their clinical manifestations and prognosis, such as a lower incidence of chest pain in young women, a higher incidence of other symptoms, and a higher mortality rate during hospitalization.^31^ It's worth noting that both OSA and MetS have sex differences, such as OSA is often seen as a male disease, while MetS may be more prevalent in females.^17^ Given that sex affects on the association among ACS, OSA, and MetS, it is fundamental to discuss and evaluate the relationship between OSA comorbid in MetS and adverse cardiovascular outcomes in ACS patients by sex. Interestingly, our study first found that ACS patients comorbid with MetS and OSA were associated with an increased risk of subsequent cardiovascular events only in women but not in men, suggesting that female ACS patients with OSA and MetS should be paid enough attention in diagnosis and treatment.

Specifically, comorbid OSA increased recurrent cardiovascular risk in female ACS patients with MetS, and this might be explained by gender differences in the pathophysiology, clinical symptoms, and disease manifestations of OSA. OSA is known to be more prevalent in men than women, but its prevalence increases in postmenopausal women, mainly due to loss of sex hormone protection in women and a similar disruption of insulin secretion and action.^32^ Postmenopausal estrogen loss is associated with obesity, cardiovascular disease, and elevated markers of inflammation.^32^ In addition, endocrine dysfunction such as hypothyroidism is very common in women, which itself may induce OSA.^33^ Moreover, insulin sensitivity is different in gender that systemic insulin sensitivity is higher in women than men.^7^ Women with OSA typically manifest as a clustering of apnea during rapid eye movement (REM) sleep, which may be associated with nocturnal ischemia and increased nocturnal sympathetic activity, resulting in a higher risk of cardiovascular events.^34^ The phenotype of female OSA is closely related to age, hypertension, and abdominal obesity.^35^ Women also had a higher risk of high‐sensitivity troponin T, endothelial dysfunction, and heart failure or death associated with OSA compared with men.^6^, ^36^

Currently, data from controlled trials failed to find a positive effect of CPAP treatment on cardiovascular outcomes.^37^, ^38^, ^39^ Those negative results might partly be explained by the heterogeneity of ACS patients and mean that OSA might only exert deleterious synergistic effects in the high‐risk phenotype of ACS patients. Whether CPAP improves MetS is a very controversial issue. Several studies have demonstrated that long‐term and high‐adherence CPAP therapy has beneficial effects on insulin resistance, impaired glucose tolerance, and cardiac function.^17^, ^40^ Another study showed that CPAP treatment significantly increased insulin secretion and decreased circulating leptin, LDL cholesterol, and total cholesterol.^41^ In addition, a most recent study also demonstrated that 6 months of CPAP therapy in patients with OSA promoted a higher chance of MetS reversal as compared with a placebo.^42^ However, several studies found that CPAP is unlikely to have a major effect on metabolic health.^43^, ^44^

Gender difference is also associated with the effectiveness of CPAP, as men with the same severity of OSA require more OSA treatment than women.^45^ Notably, in this study, female ACS patients with combined MetS and OSA had a significantly increased risk of cardiovascular events after ACS, thus representing a high‐risk subgroup perhaps more likely to respond to the intervention. In response to the call for attention to cardiovascular disease in women and to improve the outcome of ACS, screening for OSA should not be ignored, especially when combined with MetS.

Although our study has many strengths, some limitations of this study deserve to be acknowledged. First, although the sample was large, patients with non‐MetS represented only 22.9%, and women represented only 15.5% of the overall population, which may reduce the statistical power. Women were usually less represented (14.8%–16.0%) in patients with coronary artery disease^37^, ^39^, ^46^ and patients with OSA.^47^ Second, the diagnosis of OSA in this study was based on a portable polygraph, which may overestimate actual sleep time and underestimate AHI. However, Studies have shown that portable polygraphy can be used for OSA diagnosis.^48^ Third, the prevalence and severity of OSA and MetS may vary from months to years after the onset of ACS. Therefore, data collected during hospitalization may not be representative of the severity of OSA in patients during follow‐up. Fourth, in our study, OSA was defined as an AHI ≥ 15 events/h, our grouping may lead to an increase the number of non‐OSA patients to some extent. However, this grouping method is feasible and has been validated in the previous study.^37^ Finally, this study primarily recruited East‐Asian patients, which cannot be extrapolated to patients with other ethnic or racial backgrounds.

## 5 CONCLUSIONS

In hospitalized ACS patients with MetS, comorbid OSA was associated with an increased risk of subsequent events among women. In response to the call for attention to cardiovascular disease in women, OSA screening should not be ignored in ACS patients, especially when combined with MetS.

## CONFLICT OF INTEREST STATEMENT

The authors declare no conflict of interest.

## CLINICAL TRIAL REGISTRATION

ClinicalTrials. gov; No.: NCT03362385; URL: www.clinicaltrials.gov.

## Supporting information

Supporting information.

## Data Availability

The data used in this study can be obtained from the corresponding author upon reasonable request. The data that support the findings of this study are available from the corresponding author upon reasonable request
