# Supplementary material for: Comorbid obstructive sleep apnea is associated with adverse cardiovascular outcomes in female patients with acute coronary syndrome complicating metabolic syndrome
Source: Clin Cardiol. 2023 Apr 14;46(6):663–73. doi: 10.1002/clc.24020 (PMC10270259; doi:10.1002/clc.24020)
Supplement: Supplementary file 1 — Supporting information. [file CLC-46-663-s001.docx]

**Supporting Information:**

**Figure S1. Cumulative Incidence of MACCE between MetS and non-MetS groups.**


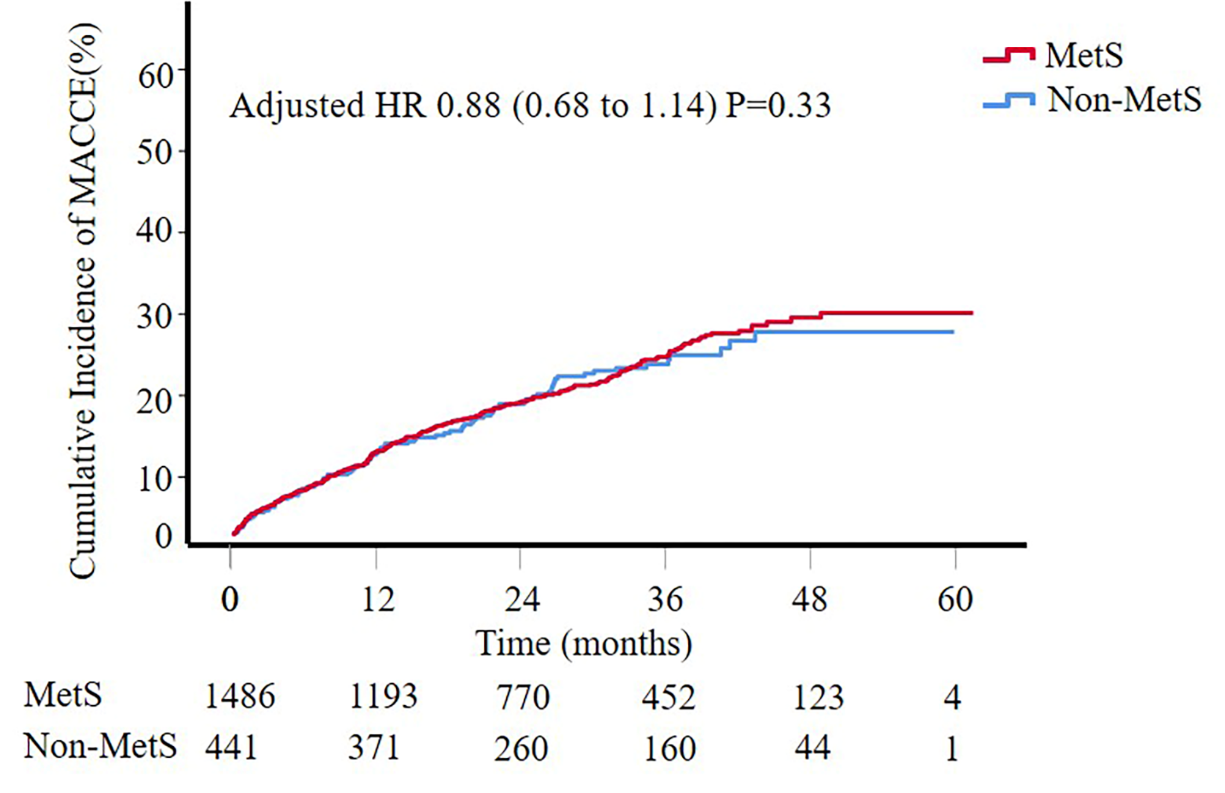


Kaplan-Meier estimates and fully-adjusted HR for MACCE between MetS and non-MetS groups. HR, hazard ratio; MetS, metabolism syndrome; OSA, obstructive sleep apnea.

**Supporting Information: Figure S2.**

**Cumulative Incidence of Hospitalization for MACCE by MetS and OSA Categories in ACS patients.**


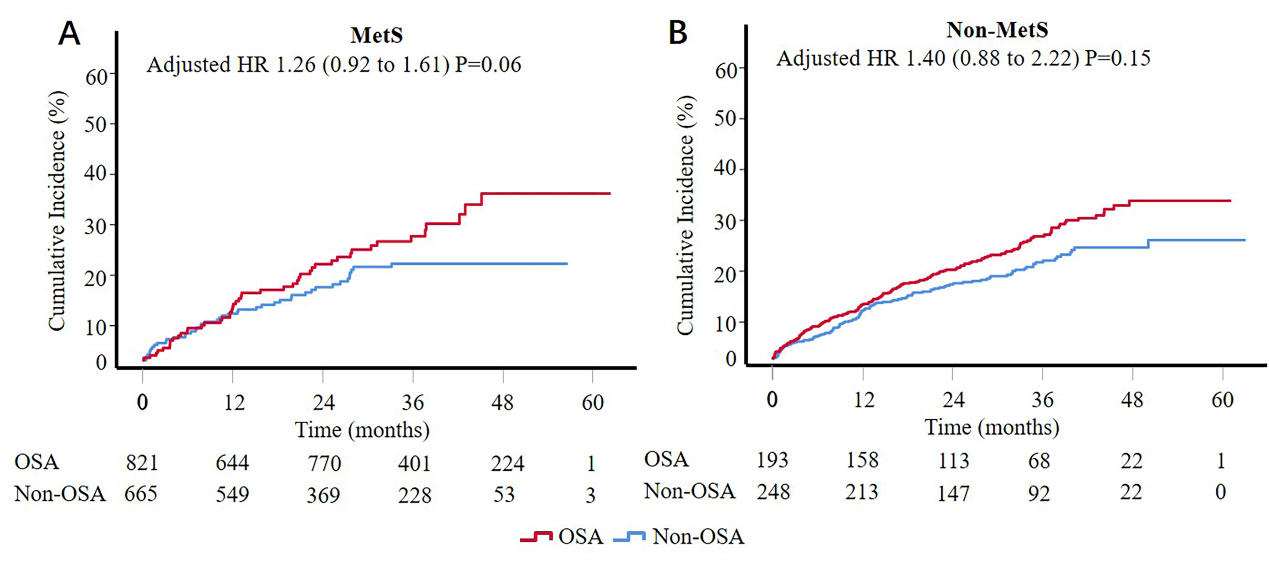


Kaplan-Meier estimates and fully-adjusted HR for MACCE between OSA and Non-OSA groups in ACS patients with MetS (A) and Non-MetS (B). HR, hazard ratio; MetS, metabolism syndrome

**Supporting Information Table S1. Baseline Clinical Characteristics in ACS patients with MetS by gender and OSA Categories**

| **Variables** | **Total (n=1486)** | **Women (n=252)** | | | **Men (n=1234)** | | |
| --- | --- | --- | --- | --- | --- | --- | --- |
|  |  | **OSA (n=108)** | **Non-OSA (n=144)** | **P value** | **OSA (n=713)** | **Non-OSA (n=521)** | **P value** |
| **Demographics** | | | | | | | |
| Age, y | 56.0±10.4 | 65.9±6.3 | 62.6±9.4 | 0.002 | 54.6±10.3 | 54.1±9.9 | 0.40 |
| BMI, kg/m^2^ | 27.6±3.5 | 27.7±3.8 | 26.2±3.81 | 0.002 | 28.5±3.3 | 26.7±3.2 | ＜0.001 |
| Waist | 101 (95-106) | 100.5 (93.8-106) | 96.0 (89.5-102.0) | 0.95 | 102 (97-109) | 99 (94-104) | ＜0.001 |
| Neck, circumference, cm | 41 (39-43) | 37.0 (36.0-39.0) | 36.0 (34.0-38.8) | 0.44 | 42 (40-44) | 41 (39-43) | 0.001 |
| Waist-to-hip ratio, median | 0.99 (0.95-1.02) | 0.97 (0.93-1.01) | 0.95 (0.92-1.00) | 0.003 | 0.99 (0.97-1.03) | 0.98 (0.95-1.02) | ＜0.001 |
| Systolic BP, mm Hg | 128 (119-139) | 130.0(120-141.8) | 130.0(120.0-143.5) | 0.74 | 128 (119-139) | 127 (117-137) | 0.26 |
| Diastolic BP, mm Hg | 72 (70-85) | 71.0 (69.25-80) | 74.0 (68.0-80.0) | 0.50 | 80 (70-87) | 76 (70-85) | ＜0.001 |
| **Medical history** | | | | | | | |
| Diabetes mellitus | 565 (38%) | 56 (51.9) | 61 (42.4) | 0.14 | 239 (33.5%) | 209 (40.1%) | 0.02 |
| Hypertension | 1067 (71.8%) | 93 (86.1) | 120 (83.3) | 0.55 | 520 (72.9%) | 334 (64.1%) | 0.001 |
| Hyperlipidemia | 530 (35.7%) | 50 (46.3) | 51 (35.4) | 0.08 | 248 (34.8%) | 113 (21.7%) | 0.99 |
| Prior stroke | 166 (11.2%) | 20 (18.5) | 13 (13.2) | 0.25 | 83 (11.6%) | 44 (8.4%) | 0.07 |
| Prior MI | 253 (17.0%) | 11 (10.2) | 13 (9.0) | 0.78 | 137 (19.2%) | 92 (17.7%) | 0.49 |
| Previous PCI | 332 (22.3%) | 22 (20.4) | 22 (15.3) | 0.29 | 175 (24.5%) | 113 (21.7%) | 0.24 |
| Previous CABG | 27 (1.8%) | 4 (3.7) | 2 (1.4) | 0.23 | 12 (1.7%) | 9 (1.7%) | 0.95 |
| Smoking | | | | 0.02 |  | | 0.45 |
| No | 527 (35.5) | 103 (95.4) | 122 (84.7) |  | 181 (25.4) | 121 (23.2) |  |
| Current | 686 (46.2) | 3 (2.8) | 16 (11.1) |  | 387 (54.3) | 280 (53.7) |  |
| Previous | 273 (18.4) | 2 (1.9) | 6 (4.2) |  | 145 (20.3) | 120 (23.0) |  |
| **Baseline tests** | | | | | | | |
| Glucose, mmol/L | 6.25 (5.57-7.98) | 6.50(5.71-8.40) | 6.11 (5.48-7.55) | 0.05 | 6.21 (5.53-8.02) | 6.25 (5.58-8.03) | 0.78 |
| Hemoglobin A1C, % | 6.20 (5.70-7.30) | 6.80 (6.10-8.15) | 6.40 (5.90-7.40) | 0.01 | 6.10 (5.70-7.20) | 6.20 (5.70-7.30) | 0.94 |
| Triglyceride, mmol/L | 1.71(1.21-2.41) | 1.62 (1.17-2.09) | 1.58 (1.19-2.21) | 0.22 | 1.75 (1.24-2.54) | 1.70 (1.20-2.50) | 0.91 |
| Total Cholesterol, mmol/L | 4.14(3.46-4.95) | 4.27 (3.64-5.17) | 4.48 (3.60-5.15) | 0.81 | 4.12 (3.45-4.87) | 4.03 (3.40-4.91) | 0.65 |
| HDL-C, mmol/L | 0.96 (0.84-1.10) | 1.10 (0.90-1.22) | 1.10 (0.96-1.26) | 0.59 | 0.93 (0.83-1.05) | 0.93 (0.82-1.06) | 0.67 |
| LDL-C, mmol/L | 2.43 (1.91-3.08) | 2.44 (1.98-3.13) | 2.57 (1.90-3.24) | 0.89 | 2.43 (1.92-3.05) | 2.38 (1.86-3.05) | 0.44 |
| Cr, μmmol/L | 73.7 (64.9-84.3) | 63.4 (55.7-74.6) | 59.7 (52.1-70.3) | 0.13 | 76.6 (67.9-86.2) | 74.8 (66.5-85.9) | 0.14 |
| LVEF, % | 62 (56-65) | 63 (59-68) | 63 (60-66) | 0.86 | 61 (55-65) | 62 (56-65) | 0.13 |
| **Diagnosis** |  |  |  | 0.02 |  |  | 0.50 |
| STEMI | 317 (21.3%) | 19 (17.6%) | 15 (10.4%) |  | 178 (25.0%) | 105 (20.2%) |  |
| NSTEMI | 286 (19.3%) | 14 (13.0%) | 27 (18.8%) |  | 141 (19.8%) | 104 (20.0%) |  |
| Unstable angina | 883 (59.4%) | 75 (69.4%) | 102 (70.8%) |  | 394 (55.3%) | 312 (59.9%) |  |
| **Sleep Study** |  |  |  |  |  |  |  |
| AHI, events/h | 17.1 (8.4-32.4) | 29.3 (20.2-39.5) | 7.7 (2.8-10.2) | ＜0.001 | 29.1 (20.8-42.7) | 7.7 (4.5-10.9) | ＜0.001 |
| ODI, events/h | 17.3 (9.5-30.1) | 27.7 (20.9-39.3) | 9.0 (4.3-12.0) | ＜0.001 | 27.5 (20.1-39.8) | 8.5 (5.0-11.8) | ＜0.001 |
| Minimum SaO_2_, % | 85 (80-88) | 82 (78-85) | 87 (84-89) | ＜0.001 | 83 (77-86) | 88 (85-90) | ＜0.001 |
| Mean SaO_2_, % | 94 (93-95) | 93 (92-94) | 94 (93-95) | ＜0.001 | 93 (92-94) | 95 (93-95) | ＜0.001 |
| Time with SaO_2_<90% | 3 (0.4-11) | 7.9 (2.9-21.2) | 1 (0.2-5) | ＜0.001 | 6 (2-15) | 0.5 (0.3-2.2) | ＜0.001 |
| Epworth Sleepiness Scale | 7.0 (4.0-11.0) | 7.0 (3.0-12.0) | 6.0 (2.0-9.0) | ＜0.001 | 9.0 (5.0-12.0) | 6.0 (4.0-11.0) | ＜0.001 |
| **Procedures** |  |  |  |  |  |  |  |
| Coronary angiography | 1447 (97.4) | 105 (97.2) | 137 (95.1) | 0.40 | 697 (97.8) | 508 (97.5) | 0.77 |
| PCI | 934 (62.9) | 62 (57.4) | 71 (49.3) | 0.20 | 479 (67.2) | 322 (61.8) | 0.052 |
| DES use | 676 (45.5) | 52 (48.1) | 65 (45.1) | 0.64 | 417 (58.5) | 276 (53.0) | 0.054 |
| CABG | 105 (7.0) | 7 (6.5) | 11 (7.6) | 0.72 | 43 (6.0) | 44 (8.4) | 0.10 |
| **Medications on discharge** |  |  |  |  |  |  |  |
| Aspirin | 1443 (97.1) | 104 (96.3%) | 139 (96.5%) | 0.92 | 693 (97.2%) | 507 (97.3%) | 0.90 |
| P2Y_12_ inhibitors | 1358 (91.4) | 98 (90.7%) | 129 (89.6%) | 0.76 | 661 (92.7%) | 470 (90.2%) | 0.18 |
| β-blockers | 1171 (78.8) | 81 (75.0%) | 111 (77.1%) | 0.70 | 574 (80.5%) | 405 (77.7%) | 0.24 |
| ACEIs/ARBs | 958 (64.5) | 75 (69.4%) | 93 (64.6%) | 0.42 | 483 (67.7%) | 307 (58.9%) | 0.001 |
| Statins | 1461 (98.3) | 104 (96.3%) | 142 (98.6%) | 0.23 | 701 (98.3%) | 514 (98.7%) | 0.63 |

Data are presented as mean ± SD, median (IQR), n (%), or n (%). ACEI, angiotensin-converting enzymes inhibitor; AHI, apnea-hypopnea index; ARB, angiotensin receptor blocker; BMI, body mass index; BP, blood pressure; CABG, coronary artery bypass grafting; Cr: Creatinine; DES: drug-eluting stent IQR, interquartile range; LVEF, left ventricular ejection fraction; MetS, metabolism syndrome; NSTEMI, non-ST-segment elevation myocardial infarction; ODI, oxygen desaturation index; OSA, obstructive sleep apnea; PCI, percutaneous coronary intervention; SD, standard deviation; STEMI, ST-segment-elevation myocardial infarction

**Supporting Information Table S2. Baseline Clinical Characteristics in ACS patients with Non-MetS by gender and OSA Categories**

| **Variables** | **Total (n=441)** | **Women (n=46)** | | | **Men (n=395)** | | |
| --- | --- | --- | --- | --- | --- | --- | --- |
|  |  | **OSA (n=34)** | **Non-OSA (n=12)** | **P value** | **OSA (n=328)** | **Non-OSA (n=67)** | **P value** |
| **Demographics** | | | | | | | |
| Age, y | 57.51±10.6 | 63.7±7.7 | 64.5±7.3 | 0.76 | 57.0±10.7 | 55.9±10.4 | 0.468 |
| BMI, kg/m^2^ | 25.3±3.6 | 26.6±3.67 | 23.7±3.34 | 0.022 | 25.6±3.7 | 23.7±2.5 | ＜0.001 |
| Waist | 98.8±10.1 | 95.3±10.5 | 88.4±8.7 | 0.07 | 94.8±10.3 | 89.3±7.4 | ＜0.001 |
| Neck, circumference, cm | 39 (37-41) | 35(34-37) | 35 (32-36) | 0.431 | 39 (37-42) | 38 (37-40) | 0.015 |
| Waist-to-hip ratio, median | 0.96 (0.92-0.99) | 0.95 (0.89-0.99) | 0.94 (0.84-0.99) | 0.741 | 0.96 (0.93-1.00) | 0.94 (0.91-0.98) | 0.001 |
| Systolic BP, mm Hg | 120 (111-132) | 129(117-140) | 130 (114-144) | 0.726 | 120 (110-130) | 120 (112-130) | 0.703 |
| Diastolic BP, mm Hg | 72 (67-80) | 72 (64-79) | 72(64-84) | 0.707 | 72 (68-80) | 71 (67-80) | 0.656 |
| **Medical history** | | | | | | | |
| Diabetes mellitus | 44 (10.0) | 3 (8.8) | 1 (8.3) | >0.999 | 34 (10.4) | 6(9.0) | 0.727 |
| Hypertension | 180 (40.8) | 20 (58.8) | 7 (58.3) | >0.999 | 130(39.6) | 23 (34.3) | 0.417 |
| Hyperlipidemia | 107(24.3) | 14(41.2) | 7 (58.3) | 0.305 | 74(22.6) | 12 (17.9) | 0.401 |
| Prior stroke | 41 (9.3) | 2 (5.9) | 1 (8.3) | >0.999 | 35 (10.7) | 3(4.5) | 0.117 |
| Prior MI | 63 (14.3) | 1(2.9) | 0 (0) | >0.999 | 51 (15.5) | 11 (16.4) | 0.859 |
| Previous PCI | 67 (15.2) | 8(23.5) | 2 (16.7) | 0.929 | 44(13.4) | 13 (19.4) | 0.204 |
| Previous CABG | 2 (0.5) | 0 (0) | 0 (0) | - | 2 (6.0) | 0 (0) | >0.999 |
| Smoking | | | | >0.999 |  | | 0.314 |
| No | 127 (28.8) | 27(79.4) | 10 (83.3) |  | 74 (22.6) | 16 (23.9) |  |
| Current | 227 (51.5)) | 7 (20.6) | 2 (16.7) |  | 186 (56.7) | 32 (47.8) |  |
| Previous | 87 (19.7) | 0 (0) | 0 (0) |  | 68 (20.7) | 19 (28.4) |  |
| **Baseline tests** | | | | | | | |
| Glucose, mmol/L | 5.3 (5.0-5.7) | 5.3(5.0-5.6) | 5.5(5.2-6.0) | 0.487 | 5.3 (5.0-5.7) | 5.2 (4.9-5.6) | 0.459 |
| Hemoglobin A1C, % | 5.7 (5.4-6.1) | 5.7 (5.5-6.2) | 5.8 (5.7-6.0) | 0.621 | 5.7(5.4-6.1) | 5.6 (5.4-5.9) | 0.018 |
| Triglyceride, mmol/L | 1.1 (0.9-1.4) | 1.2 (0.9-1.5) | 1.1(0.9-1.3) | 0.282 | 1.1 (0.8-1.4) | 1.1 (0.9-1.4) | 0.626 |
| Total Cholesterol, mmol/L | 4.1 (3.5-4.8) | 4.2 (3.8-5.2) | 3.4(3.1-4.8) | 0.071 | 4.0 (3.5 to 4.8) | 4.1 (3.4-4.8) | 0.907 |
| HDL-C, mmol/L | 1.2 (1.0-1.3) | 1.4 (1.3-1.5) | 1.4 (1.3-1.5) | 0.498 | 1.1 (1.0-1.3) | 1.2 (1.0-1.4) | 0.190 |
| LDL-C, mmol/L | 2.5(1.9-3.2) | 2.3 (1.9-3.4) | 1.7 (1.3-2.6) | 0.058 | 2.5(1.9-3.2) | 2.4 (1.8-3.1) | 0.656 |
| Cr, μmmol/L | 73.8 (64.0-82.5) | 58.4 (53.3-68.5) | 53.9 (49.2-62.2) | 0.225 | 75.2 (66.0-83.7) | 74.1 (65.8-83.4) | 0.683 |
| LVEF, % | 61 (55-66) | 63 (60-67) | 65(63-69) | 0.115 | 60 (54-65) | 64 (59 - 68) | <0.001 |
| **Diagnosis** |  |  |  | 0.253 |  |  | 0.179 |
| STEMI | 113 (25.6) | 6 (17.6) | 0 (0) |  | 95(29.0) | 12 (17.0) |  |
| NSTEMI | 79 (17.9) | 8 (23.5) | 2 (16.7) |  | 56 (17.1) | 13 (19.4) |  |
| Unstable angina | 249 (56.5) | 20 (58.8) | 10 (83.3) |  | 177 (54.0) | 42 (62.7) |  |
| **Sleep Study** |  |  |  |  |  |  |  |
| AHI, events/h | 12.1 (6.4-25.2) | 23.1 (9.1-39.0) | 2.1 (1.3-3.4) | ＜0.001 | 16.1 (9.3-27.4) | 2.3 (1.3-3.3) | ＜0.001 |
| ODI, events/h | 12.6 (7.3-24.3) | 24.1 (12.4-39.1) | 4.0 (3.1-6.1) | ＜0.001 | 14.8 (9.9-25.2) | 3.1 (1.7-4.2) | ＜0.001 |
| Minimum SaO_2_, % | 87 (83-89) | 84 (78-88) | 89(87-91) | 0.001 | 86 (82-88) | 90 (87-92) | ＜0.001 |
| Mean SaO_2_, % | 94 (93-95) | 93 (92-95) | 95 (94-96) | 0.017 | 94 (93-95) | 95 (94-96) | ＜0.001 |
| Time with SaO_2_<90% | 1.0 (0.2-5.8) | 5.9(0.8-10.9) | 0.4(0-1.0) | 0.001 | 1.2(0.4-6.0) | 0.0 (0.0-1.0) | ＜0.001 |
| Epworth Sleepiness Scale | 7.0(3.0-11.0) | 6.0 (4.0-9.0) | 8.0 (4.0-10.0) | 0.334 | 7.0 (3.0-11.0) | 7.0 (3.0-10.0) | 0.934 |
| **Procedures** |  |  |  |  |  |  |  |
| Coronary angiography | 430(97.5) | 34 (100) | 12 (100) | - | 318 (97.0) | 66 (98.5) | 0.766 |
| PCI | 275 (62.4) | 17 (50.0) | 7 (58.3) | 0.619 | 209 (63.7) | 42 (62.7) | 0.873 |
| DES use | 241 (54.6) | 16(47.1) | 5 (41.7) | 0.747 | 183 (55.8) | 37(55.2) | 0.932 |
| CABG | 25 (5.7) | 1 (2.9) | 0(0) | >0.999 | 20(6.1) | 4 (6.0) | >0.999 |
| **Medications on discharge** |  |  |  |  |  |  |  |
| Aspirin | 437(98.4) | 33 (97.1) | 11 (91.7) | 0.458 | 323 (98.5) | 0 (0) | 0.594 |
| P2Y_12_ inhibitors | 410(93.0) | 33 (97.1) | 9 (75.0) | 0.083 | 305 (93.0) | 63 (94.0) | 0.966 |
| β-blockers | 317 (71.9) | 27(79.4) | 7(58.3) | 0.295 | 236 (72.0) | 47(70.1) | 0.766 |
| ACEIs/ARBs | 237 (53.7) | 21 (61.8) | 3(25.0) | 0.028 | 179 (54.6) | 34(50.7) | 0.567 |
| Statins | 436(98.9) | 34 (100) | 11 (91.7) | 0.261 | 324 (98.8) | 67 (100) | >0.999 |

Data are presented as mean ± SD, median (IQR), n (%), or n (%). ACEI, angiotensin-converting enzymes inhibitor; AHI, apnea-hypopnea index; ARB, angiotensin receptor blocker; BMI, body mass index; BP, blood pressure; CABG, coronary artery bypass grafting; Cr: Creatinine; DES: drug-eluting stent IQR, interquartile range; LVEF, left ventricular ejection fraction; MetS, metabolism syndrome; NSTEMI, non-ST-segment elevation myocardial infarction; ODI, oxygen desaturation index; OSA, obstructive sleep apnea; PCI, percutaneous coronary intervention; SD, standard deviation; STEMI, ST-segment-elevation myocardial infarction.

**Supporting Information: Table S3. Cox Regression Analyses Evaluating the Association Between MetS and Non-MetS groups of the Risk of Cardiovascular Events.**

| **Variables** | **Unadjusted HR**  **(95% CI)** | **P value** | **Adjusted HR***  **(95% CI)** | **P value** |
| --- | --- | --- | --- | --- |
| MACCE | 1.04 (0.83-1.32) | 0.72 | 0.88 (0.68-1.14) | 0.33 |
| Cardiovascular death | 1.40 (0.58-3.40) | 0.45 | 1.46 (0.55-3.84) | 0.44 |
| Myocardial infarction | 1.33 (0.67-2.65) | 0.42 | 1.05 (0.49-2.24) | 0.91 |
| Stroke | 1.04 (0.52-2.12) | 0.91 | 0.74 (0.33-1.62) | 0.45 |
| Ischemia-driven revascularization | 1.08 (0.74-1.56) | 0.70 | 0.97 (0.65-1.46) | 0.89 |
| Hospitalization for unstable angina | 1.23 (0.93-1.62) | 0.14 | 0.92 (0.67-1.25) | 0.59 |
| Hospitalization for heart failure | 1.04 (0.79-1.38) | 0.77 | 0.90 (0.29-2.82) | 0.86 |
| Composite for cardiovascular death, myocardial infarction, or ischemic stroke | 1.24 (0.80-1.93) | 0.34 | 0.95 (0.59-1.55) | 0.85 |
| Composite for cardiac events | 1.08 (0.84-1.39) | 0.54 | 0.92 (0.70-1.21) | 0.10 |
| All‑cause death | 1.13 (0.56-2.28) | 0.73 | 0.97 (0.44-2.11) | 0.94 |
| All repeat revascularization | 1.14 (0.83-1.56) | 0.43 | 1.05(0.75-1.49) | 0.77 |

Data are presented as median (IQR). Comparison between MetS and Non-MetS groups. *Model adjusted for age, sex, body mass index, smoking, hypertension, diabetes mellitus, hyperlipidemia, prior myocardial infarction, prior stroke, and clinical presentation (acute myocardial infarction vs unstable angina). Composite for cardiac events (cardiovascular death, myocardial infarction, ischemia-driven revascularization, or hospitalization for unstable angina or heart failure); CI, confidence interval; HR, hazard ratio; MACCE, major adverse cardiovascular and cerebrovascular event; MetS, metabolism syndrome; OSA, obstructive sleep apnea.

**Supporting Information: Table S4. Crude Number of all Events by MetS and OSA Categories in ACS Patients.**

| **Variables** | **MetS (n=1486)** | **Non-MetS (n=441)** | **OSA (n=1014)** | **Non-OSA(n=913)** |
| --- | --- | --- | --- | --- |
| MACCE | 299 (20.1) | 90 (20.4) | 227 (22.4) | 162 (17.7) |
| Cardiovascular death | 27 (1.8) | 6 (1.4) | 19 (1.9) | 14 (1.5) |
| Myocardial infarction | 41 (2.8) | 10 (2.3) | 33 (3.3) | 18 (2.0) |
| Stroke | 33 (2.2) | 10 (2.3) | 25 (2.5) | 18 (2.0) |
| Ischemia-driven revascularization | 123 (8.3) | 36 (8.2) | 94 (9.3) | 65 (7.1) |
| Hospitalization for unstable angina | 209 (14.1) | 63 (14.3) | 155 (15.3) | 117 (12.8) |
| Hospitalization for heart failure | 16 (1.1) | 5 (1.1) | 11 (1.1) | 10 (1.1) |
| Composite of major cardiovascular events | 97 (6.5) | 25 (5.7) | 76 (7.5) | 46 (5.0) |
| Composite for cardiac events | 273 (18.4) | 80 (18.1) | 205 (20.2) | 148 (16.2) |
| All repeat revascularization | 178 (12.0) | 49 (11.1) | 129 (12.7) | 98 (10.7) |
| All death | 36 (2.4) | 10 (2.3) | 23 (2.3) | 23 (2.5) |

Data are presented as n (%). Composite end point of major cardiovascular events included cardiovascular death, myocardial infarction, and stroke; Composite for cardiac events included cardiovascular death, myocardial infarction, ischemia-driven revascularization, or hospitalization for unstable angina or heart failure. CI, confidence interval; MACCE, major adverse cardiovascular and cerebrovascular event; MetS, metabolism syndrome; OSA, obstructive sleep apnea.

**Supporting Information: Table S5. Crude Number of all Events in ACS patients with MetS by Sex and OSA Categories.**

|  | **Women (n=252)** | | **Men (n=1234)** | |
| --- | --- | --- | --- | --- |
| **Variables** | **OSA (n=108)** | **Non-OSA (n=144)** | **OSA (n=713)** | **Non-OSA (n=521)** |
| MACCE | 30 (27.8%) | 26 (18.1%) | 150 (21.0%) | 93 (17.9%) |
| Cardiovascular death | 2 (1.9%) | 1 (0.7%) | 15 (2.1%) | 9 (1.7%) |
| Myocardial infarction | 4 (3.7%) | 2 (1.4%) | 23 (3.2%) | 12 (2.3%) |
| Stroke | 2 (1.9%) | 6 (4.2%) | 17 (2.4%) | 8 (1.5%) |
| Ischemia-driven revascularization | 12 (11.1%) | 9 (6.3%) | 64 (9.0%) | 38 (7.3%) |
| Hospitalization for unstable angina | 25 (23.1%) | 18 (12.5%) | 98 (13.7%) | 68 (13.1%) |
| Hospitalization for heart failure | 0 | 2 (1.4%) | 7 (1.0%) | 7 (1.3%) |
| Composite of major cardiovascular events | 8 (7.4%) | 9 (6.3%) | 54 (7.6%) | 26 (5.0%) |
| Composite for cardiac events | 28 (25.9%) | 22 (15.3%) | 136 (19.1%) | 87 (16.7%) |
| All repeat revascularization | 15 (13.9%) | 10 (6.9%) | 88 (12.3%) | 65 (12.5%) |
| All death | 4 (3.7%) | 4 (2.8%) | 16 (2.2%) | 12 (2.3%) |

Composite end point of major cardiovascular events included cardiovascular death, myocardial infarction, and stroke; Composite for cardiac events included cardiovascular death, myocardial infarction, ischemia-driven revascularization, or hospitalization for unstable angina or heart failure. CI, confidence interval; MACCE, major adverse cardiovascular and cerebrovascular event; OSA, obstructive sleep apnea.

**Supporting Information: Table S6. Cox Regression Analyses Evaluating the Association Between OSA and Risk of Cardiovascular Events by Sex in Patients with ACS and Non-MetS.**

|  | **Women (n=46)** | | | | **Men (n=395)** | | | |
| --- | --- | --- | --- | --- | --- | --- | --- | --- |
| **Variables** | **Unadjusted HR**  **(95% CI)** | **P value** | **Adjusted HR***  **(95% CI)** | **P value** | **Unadjusted HR**  **(95% CI)** | **P value** | **Adjusted HR***  **(95% CI)** | **P value** |
| MACCE | 1.32 (0.43-4.10) | 0.63 | 1.78 (0.43-7.32) | 0.43 | 1.46 (0.94-2.28) | 0.09 | 1.37 (0.83-2.25) | 0.22 |
| Cardiovascular death**†** | - | - | - | - | 0.62 (0.11-3.41) | 0.59 | 0.22 (0.03-1.87) | 0.22 |
| Myocardial infarction**†** | 0.99 (0.06-16.2) | 0.99 | - | - | 2.15 (0.51-8.98) | 0.30 | 1.23 (0.22-6.85) | 0.82 |
| Stroke**†** | 1.31 (0.08-21.1) | 0.85 | - | - | 2.13 (0.51-8.92) | 0.30 | 3.48 (0.66-18.5) | 0.14 |
| Ischemia-driven revascularization**†** | 7.36 (0.86-63.0) | 0.07 | - | - | 0.99 (0.48-2.04) | 0.98 | 0.69 (0.31-1.58) | 0.38 |
| Hospitalization for unstable angina | 1.75 (0.47-6.51) | 0.41 | 2.59 (0.53-12.7) | 0.24 | 1.32 (0.77-2.25) | 0.31 | 1.25 (0.70-2.24) | 0.46 |
| Hospitalization for heart failure**†** | - | - | - |  | 4.81 (0.54-43.1) | 0.16 | - | - |
| Composite for cardiovascular death, myocardial infarction, or ischemic stroke**†** | 1.12 (0.16-8.00) | 0.91 | - | - | 1.70 (0.72-4.03) | 0.23 | 1.38 (0.48-3.93) | 0.55 |
| Composite for cardiac events | 1.34 (0.39-4.62) | 0.65 | 1.94 (0.44-8.54) | 0.38 | 1.39 (0.87-2.22) | 0.17 | 1.25 (0.74-2.11) | 0.40 |
| All‑cause death**†** | - | - | - |  | 0.63 (0.16-2.53) | 0.52 | 0.21 (0.03-1.27) | 0.09 |
| All repeat revascularization**†** | - | - | - | - | 1.18 (0.64-2.16) | 0.60 | 1.05 (0.54-2.05) | 0.89 |

Data are presented as median (IQR). *****Model adjusted for age, sex, body mass index, smoking, hypertension, diabetes mellitus, hyperlipidemia, prior myocardial infarction, prior stroke, and clinical presentation (acute myocardial infarction vs unstable angina). **†** Univariate and multivariate Cox regression was not done due to few numbers of events; Composite for cardiac events (cardiovascular death, myocardial infarction, ischemia-driven revascularization, or hospitalization for unstable angina or heart failure); CI, confidence interval; HR, hazard ratio; MACCE, major adverse cardiovascular and cerebrovascular event; MetS, metabolism syndrome; OSA, obstructive sleep apnea.

**Supporting Information: Table S7. Crude Number of all Events in ACS patients with Non-MetS by Sex and OSA Categories.**

|  | **Women (n=46)** | | **Men (n=395)** | |
| --- | --- | --- | --- | --- |
| **Variables** | **OSA (n=20)** | **Non-OSA (n=26)** | **OSA (n=173)** | **Non-OSA (n=222)** |
| MACCE | 6 (30.0) | 26 (23.1) | 41 (23.7) | 37 (16.7) |
| Cardiovascular death | 0 (0) | 0 (0) | 2 (1.2) | 4 (1.8) |
| Myocardial infarction | 1 (5.0) | 1 (3.8) | 5 (2.9) | 3 (1.4) |
| Stroke | 1 (5.0) | 1 (3.8) | 5 (2.9) | 3 (1.4) |
| Ischemia-driven revascularization | 5 (25.0) | 1 (3.8) | 13 (7.5) | 17 (7.7) |
| Hospitalization for unstable angina | 5 (25.0) | 4 (15.4) | 27 (15.6) | 27 (12.2) |
| Hospitalization for heart failure | 0 (0) | 0 (0) | 4 (2.3) | 1 (0.5) |
| Composite of major cardiovascular events | 2 (10.0) | 2 (7.7) | 12 (6.9) | 9 (4.1) |
| Composite for cardiac events | 5 (25.0) | 5 (19.2) | 36 (20.8) | 34 (15.3) |
| All repeat revascularization | 6 (30.0) | 1 (3.8) | 20 (11.6) | 22 (9.9) |
| All death | 0 (0) | 1 (3.8) | 3 (1.7) | 6 (2.7) |

Data are presented as n (%). Composite end point of major cardiovascular events included cardiovascular death, myocardial infarction, and stroke; Composite for cardiac events included cardiovascular death, myocardial infarction, ischemia-driven revascularization, or hospitalization for unstable angina or heart failure. CI, confidence interval; MACCE, major adverse cardiovascular and cerebrovascular event; MetS, metabolism syndrome; OSA, obstructive sleep apnea.
